# Multi-Material Radial Phononic Crystals to Improve the Quality Factor of Piezoelectric MEMS Resonators

**DOI:** 10.3390/mi15010020

**Published:** 2023-12-22

**Authors:** Qian Yang, Tianhang Gao, Chuang Zhu, Lixia Li

**Affiliations:** School of Mechanical and Electrical Engineering, Xi’an University of Architecture and Technology, Xi’an 710055, China; yangqian@xauat.edu.cn (Q.Y.); gaoth010109@163.com (T.G.); 18191215327@163.com (C.Z.)

**Keywords:** radial phononic crystal, piezoelectric MEMS resonator, anchor loss, quality factor, band gap

## Abstract

In this paper, a multi-material radial phononic crystal (M-RPC) structure is proposed to reduce the anchor-point loss of piezoelectric micro-electro-mechanical system (MEMS) resonators and improve their quality factor. Compared with single-material phononic crystal structures, an M-RPC structure can reduce the strength damage at the anchor point of a resonator due to the etching of the substrate. The dispersion curve and frequency transmission response of the M-RPC structure were calculated by applying the finite element method, and it was shown that the M-RPC structure was more likely to produce a band-gap range with strong attenuation compared with a single-material radial phononic crystal (S-RPC) structure. Then, the effects of different metal–silicon combinations on the band gap of the M-RPC structures were studied, and we found that the largest band-gap range was produced by a Pt and Si combination, and the range was 84.1–118.3 MHz. Finally, the M-RPC structure was applied to a piezoelectric MEMS resonator. The results showed that the anchor quality factor of the M-RPC resonator was increased by 33.5 times compared with a conventional resonator, and the insertion loss was reduced by 53.6%. In addition, the loaded and unloaded quality factors of the M-RPC resonator were improved by 75.7% and 235.0%, respectively, and at the same time, there was no effect on the electromechanical coupling coefficient.

## 1. Introduction

Micro-electro-mechanical system (MEMS) technology shows good promise in wireless communication systems and sensor networks [1,2,3]. In MEMS resonator-based applications, a high quality factor (Q) is ideal for realizing high-resolution sensors and low-insertion-loss filters [4,5]. Anchor loss is a key factor affecting the Q of a resonator [4,6,7,8]. Therefore, it is of great significance to suppress anchor loss and improve the Q of a resonator for practical applications [9].

Due to the significant characteristics of band gaps in phononic crystals (PnCs), acoustic wave propagation and mechanical vibration are prevented within their band-gap ranges [10]. Therefore, using PnCs to attenuate the energy leaked by an anchor point has become a study hotspot [11,12,13,14,15,16,17,18,19]. Ardito et al. improved the anchor-loss quality factor (Q_anchor_) from 344 to 105,900 by optimizing the shapes of PnCs [11]. In 2019, Bao et al. proposed a spider web PnC structure, and its Q_anchor_ was increased from 5870 to 64,800 [12]. Workie et al. studied a prism-shaped PnC and found that the unloaded quality factor (Q_u_) of a traditional resonator was increased by 1.7 times (for a butterfly rounded-edge resonant structure), 2.78 times (for a PnC deployed on the tether), and 2.8 times (for a PnC deployed on the anchor), respectively [13]. In 2022, Li et al. investigated a double “I” -hole PnC, and the results showed that the Q_anchor_ of the resonator was 20,425.1% higher than that of a traditional resonator [14]. In 2023, Awad et al. designed a reem-shaped PnC and deployed it on the anchored boundary of a resonator, resulting in an increase of 33.3 times in the Q_anchor_ value and 1.2 times in the Q_u_ value, respectively [15]. Meanwhile, studies about small cross-section connected shapes [16], solid disk shapes [17], snowflake shapes [18], and cross-shaped PnCs [19] have been reported. The different PnCs described above improved the Q of resonators while the PnCs studied were based on the band-gap characterization carried out in the Cartesian coordinate system.

Radial phononic crystals (RPCs) are cyclic structures periodically arranged along the radial direction based on cylindrical coordinate system, and they have complete band-gap characteristics [20,21,22,23,24,25,26,27,28]. Torrent et al. studied the propagation characteristics of body waves in RPCs and found that the propagation of body waves in specific frequency bands were prohibited, and they verified the band-gap characteristics [20,21,22]. Ma et al. investigated the Lamb wave propagation characteristics in a double-layer RPC plate and explained the band-gap mechanism [23]. Shu et al. proposed a piezoelectric RPC and studied the band-gap propagation behavior [24]. Shu et al. proposed two types of PnCs with uniform materials arranged radially, and the main band-gap formation mechanism was attributed to the Bragg scattering effect [25]. Li et al. proposed one-dimensional binary RPC plates, which could generate lower and wider acoustic band gaps [26]. An et al. proposed a two-dimensional cylindrical shell structure with radial and circumferential cycles, and they investigated the band-gap characteristics of the radial propagation in the inner ring of the structure, finding that the radial waves had significant attenuation within a certain frequency [27]. The RPCs designed by the above researchers have excellently achieved isolating acoustic waves. In the field of MEMS resonators, He et al. proposed an H-shaped RPC structure for a Lamb-wave resonator based on a cylindrical coordinate system [28], which generated a band gap and improved the Q of the resonator. Since the studied structure was a single material, this caused some strength damage to the resonator at the anchor point when etching the substrate, which resulted in poor stability of the structure.

In order to solve this problem, this paper proposes an “L”-type multi-material radial phononic crystal (M-RPC) structure. The M-RPC structure consisted of an “L” -shaped planar rotation. In the second part, the radial phononic crystal model and the theoretical method for calculating the dispersion curve are introduced. In the third part, the finite element method used to calculate the dispersion curve and frequency transmission response is described, and the effects of different metal–silicon combinations and structural parameters on the band gap are discussed. In the fourth part, the M-RPC structure applied to a piezoelectric MEMS resonator to analyze and compare the performance parameters in vibration mode is discussed. Finally, the entire paper is summarized.

## 2. Materials and Methods

### 2.1. Radial Phononic Crystal Model

**Figure 1:**
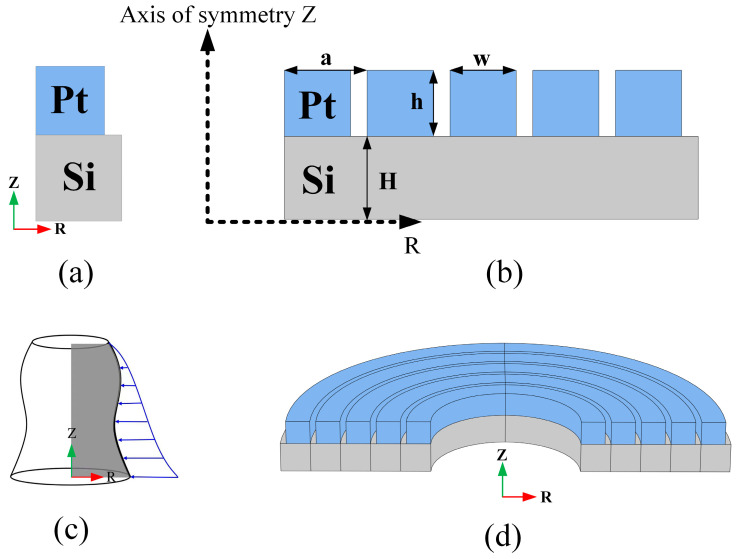
(**a**) M-RPC unit cell. (**b**) Schematic diagram of the RZ direction cross-section of the M-RPC. (**c**) The formation of the M-RPC. (**d**) A three-dimensional model formed by the five-cycle M-RPC rotation of 180°.

In this study, an anisotropic silicon plate was covered with a metal material to form an “L” shape, and it was rotated around the symmetry axis to form an M-RPC structure, as shown in Figure 1. Figure 1a shows the cell cross-section of the M-RPC, Figure 1b shows the RZ direction cross-section of the five-cycle M-RPC, Figure 1c shows the lattice formation of the M-RPC, and Figure 1d shows a three-dimensional schematic diagram of the M-RPC structure. The different colors indicate the different materials, and blue represents Pt and gray represents anisotropic Si. The lattice constant was equal to 10 µm, the width w and height h of the metal material were both 8 µm, and the thickness of the Si plate H was equal to 10 µm.

### 2.2. Theoretical Method

In order to study the propagation of the Lamb waves and subwavelength band-gap characteristics in the RPCs, a finite element method was used to calculate the dispersion curves and displacement fields, and this method has been demonstrated in the literature [23]. The M-RPC studied in this paper adopted a two-dimensional axisymmetric finite element method based on a cylindrical coordinate system to study the structural band-gap characteristics. The elastic wave equation in the cylindrical coordinate system is given by Equation (1), as follows:

(1) $$\begin{array}{l} \rho \frac{\partial^{2}u}{\partial t^{2}}=\left(\lambda +2\mu \right)\frac{\partial \theta_{t}}{\partial r}-\frac{2\mu }{r}\frac{\partial {w^{\prime }}_{z}}{\partial \theta }+2\mu \frac{\partial {w^{\prime }}_{\theta }}{\partial z}\\ \rho \frac{\partial^{2}v}{\partial t^{2}}=\left(\lambda +2\mu \right)\frac{\partial \theta_{t}}{r\partial \theta }-2\mu \frac{\partial {w^{\prime }}_{z}}{\partial z}+2\mu \frac{\partial {w^{\prime }}_{r}}{\partial r}\\ \rho \frac{\partial^{2}w}{\partial t^{2}}=\left(\lambda +2\mu \right)\frac{\partial \theta_{t}}{\partial z}-\frac{2\mu }{r}\frac{\partial }{\partial r}\left(r{w^{\prime }}_{\theta }\right)+\frac{2\mu }{r}\frac{\partial {w^{\prime }}_{r}}{\partial \theta } \end{array},$$

where u , v , and w are the displacement components in the cylindrical coordinate system, ρ is the density, t is the time, λ and μ are the elastic wave constants of the material, and r , θ , and z represent the coordinate components in a cylindrical coordinate system, respectively. In addition, the volumetric strain $\theta_{t}$ and $\left({w^{\prime }}_{r},{w^{\prime }}_{\theta },{w^{\prime }}_{z}\right)$ rotational component are defined as follows:

(2) $$\begin{array}{l} \theta_{t}=\frac{1}{r}\frac{\partial \left(ru\right)}{\partial r}+\frac{1}{r}\frac{\partial v}{\partial \theta }\frac{\partial w}{\partial z},\;{w^{\prime }}_{r}=\frac{1}{2}\left(\frac{1}{r}\frac{\partial w}{\partial \theta }+\frac{1}{r}\frac{\partial v}{\partial z}\right)\\ {w^{\prime }}_{\theta }=\frac{1}{2}\left(\frac{1}{r}\frac{\partial u}{\partial \theta }+\frac{\partial w}{\partial r}\right),{w^{\prime }}_{z}=\frac{1}{2}\left(\frac{1}{r}\frac{\partial \left(rv\right)}{\partial \theta }+\frac{\partial u}{\partial \theta }\right) \end{array}.$$

Due to the periodicity of the RPC structure in the radial direction, according to Bloch’s theorem, only one lattice unit needed to be studied, and the boundary condition equation of the lattice was established as follows:

(3) $$\mu \left(r+r_{a},z\right)=\mu \left(r,z\right)e^{ik_{r}r_{a}},$$

where r represents the radial position, $r_{a}$ represents the lattice constant, and $k_{r}$ is defined as the wave vector along the radial direction.

## 3. Band-Gap Characteristics of the RPCs

### 3.1. Phononic Crystal Band Gap

**Figure 2:**
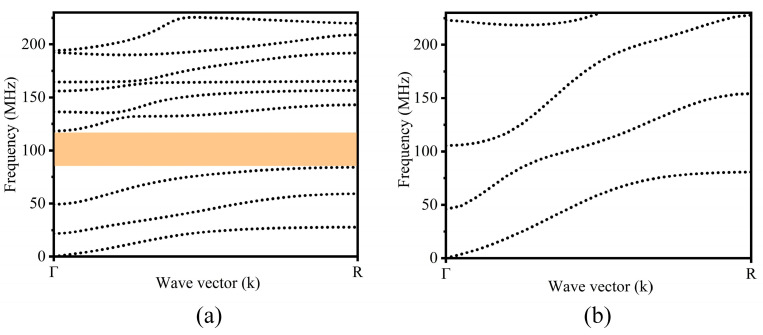
(**a**) Dispersion curve of the “L”-type M-RPC structure comprised of a Pt and Si combination. The orange area in the figure shows the band gap range. (**b**) Dispersion curve of the “L”-type S-RPC structure comprised of an Si and Si combination.

To study the propagation characteristics of the Lamb waves in the proposed “L”-type RPC structures, the dispersion curves of the “L”-type M-RPC structure (the Pt and Si combination) and the “L”-type S-RPC structure (an Si and Si combination) in the range of 0–230 MHz were calculated using COMSOL Multiphysics 6.0 finite element simulation software, as shown in Figure 2. In Figure 2a, it can be observed that the “L”-type M-RPC structure composed of Pt and Si produced a complete band gap at 84.1–118.3 MHz. As seen in Figure 2b, the S-RPC structure composed of Si and Si did not generate a band gap. From Figure 2, it can be seen that compared with the “L”-type S-RPC structure composed of Si and Si, the “L”-type M-RPC structure composed of Pt and Si was more prone to band gaps.

### 3.2. Frequency Response

**Figure 3:**
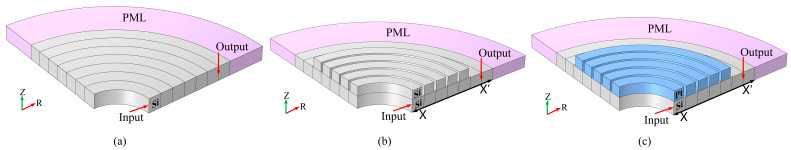
The delay line model. (**a**) Control group delay line. (**b**) Five-cycle S-RPC delay line. (**c**) Five-cycle M-RPC delay line.

In order to verify the stopband effect in radial phononic crystal structures with finite arrays, three different delay line models were established for the comparative analysis, as shown in Figure 3. Figure 3a shows the control group delay line model, Figure 3b shows the “L”-type S-RPC delay line model, and Figure 3c shows the “L”-type M-RPC delay line model. A line displacement excitation was applied to one side of the delay line model, and the other end picked up the line displacement response results. A perfect matching layer (PML) was placed at the edges of the model to eliminate the effect of boundary reflections on the results.

**Figure 4:**
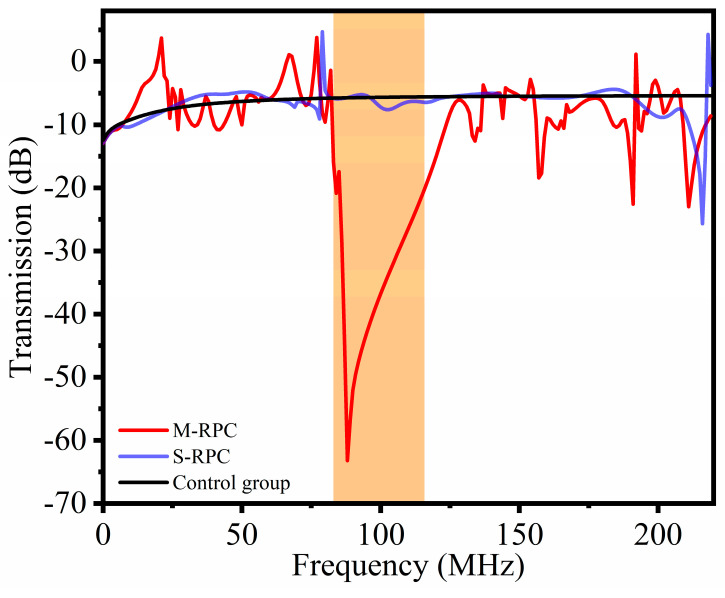
Frequency response curves of the delay line model. The solid black line is the control group delay line, the solid blue line is the S−RPC delay line, and the solid red line is the M−RPC delay line. The orange area in the figure shows the attenuation in the band gap range.

Figure 4 shows the frequency response curves of the three different delay line models. In the range of 84.1–118.3 MHz, compared to the control group delay line model and the S-RPC delay line model, the M-RPC delay line model had good attenuation, which was consistent with the band gap range calculated in the dispersion curve. Moreover, when the frequency was 88 MHz, the M-RPC delay line model reached a maximum attenuation of −63 dB.

**Figure 5:**
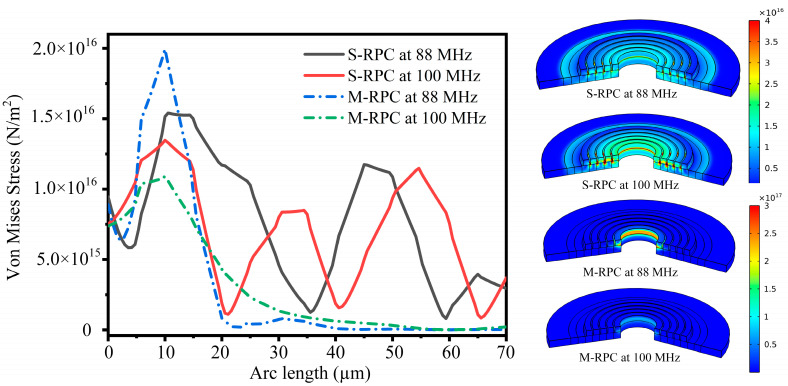
Stress plots along X-X’ at 88 MHz and 100 MHz for the S-RPC delay line model and the M-RPC delay line model.

In order to study the propagation characteristics of the Lamb waves in the radial direction using the S-RPC delay line model and the M-RPC delay line model at specific frequencies, 88 MHz and 100 MHz were selected as the research objects. This was because 88 MHz is the frequency value corresponding to the maximum attenuation in Figure 4, and 100 MHz was used as the resonant frequency for the subsequent resonator design. Figure 5 shows the stress diagram of the S-RPC delay line model and the M-RPC delay line model along X-X’ (see Figure 3). Observing Figure 5, it can be seen that at 88 MHz and 100 MHz, the M-RPC delay line model with the Pt and Si combination exhibited maximum stress values at the first lattice. As the radial distance increased, the stress values exhibited exponential decay. This was because both 88 MHz and 100 MHz were within the band gap range of the M-RPC, and the propagation of the acoustic waves was prohibited in the band-gap range, which belonged to the local resonance mechanism in phononic crystals. This mechanism can also be seen in the pattern presented by the frequency decay curves shown in Figure 4 [29]. However, for the S-RPC structure without a band gap, at 88 MHz and 100 MHz, the stress values appeared to decrease with the increase in the radial distance, and this was because the area of the radial phononic crystal structure was increasing with the increase in the number of periods of the radial structure, and so the stress values per unit area decreased.

### 3.3. The Influence of the Materials on the Band-Gap Structures

**Figure 6:**
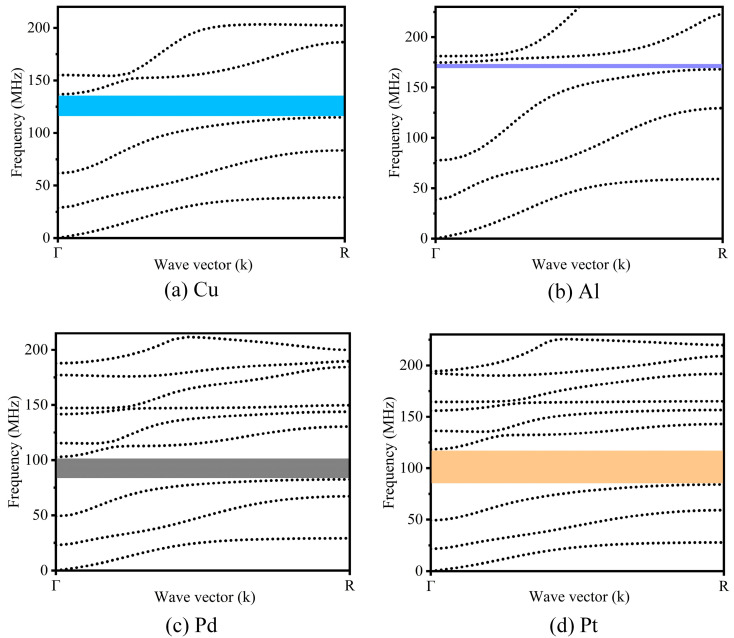
Dispersion curves of the “L”-type radial phononic crystals composed of the different metal materials and Si combinations. The blue region represents the band gap of Cu combined with Si, the purple part represents the band gap of Al combined with Si, the black part represents the band gap of Pd combined with Si, and the orange part represents the band gap of Pt combined with Si.

Different materials can have an impact on the band-gap width of a structure. For a solid–solid PnC, the mass density and the Young’s modulus are the two main factors that influence the band gap. In this study, four different metals (copper, aluminum, lead, and platinum) were covered on the silicon plate, and the specific parameters of the four materials are given in Table 1. Among them, Cu and Si have similar Young’s moduli, but the mass density of Cu is quite different from that of Si. Al and Si have significantly different Young’s moduli, but the difference in their mass densities is not significant. Pd has a similar Young’s modulus to that of Al, but the difference in the mass density between Pd and Si is quite large. The Young’s modulus of Pt is similar to that of Si, but its mass density is very different from that of Si. Figure 6 depicts the dispersion curves of the PnCs composed of silicon and the different metal materials. Figure 6a shows a multi-material phononic crystal composed of copper and silicon, and its band gap range was 114.8–136.7 MHz. Figure 6b shows that the combination of aluminum and silicon produced a narrow band gap of 168.0 to 174.8 MHz in the multi-material phononic crystal. As shown in Figure 6c, a multi-material phononic crystal composed of lead and silicon generated a complete band gap that ranged from 82.5 to 102.7 MHz. As shown in Figure 6d, the band gap of the platinum–silicon combined multi-material phononic crystal extended from 84.1 MHz to 118.3 MHz. Among the four band gaps mentioned above, the multi-material radial phononic crystal structure of the metal Pt produced the widest band gap. Observing Figure 6, it can be seen that the differences in the material parameters between the metal and silicon increased, and specifically, as the differences between the mass densities and Young’s moduli of the metal and the Si increased, the band-gap width also increased. Generally, a larger band gap provides a better performance in preventing acoustic wave propagation [13]. In addition, Pt is a CMOS (complementary metal oxide semiconductor) -compatible metal. Therefore, Pt was the first choice as the research material for the phononic crystal resonator in this study.

### 3.4. The Effect of Metal Height and Width on the Band-Gap Structure

**Figure 7:**
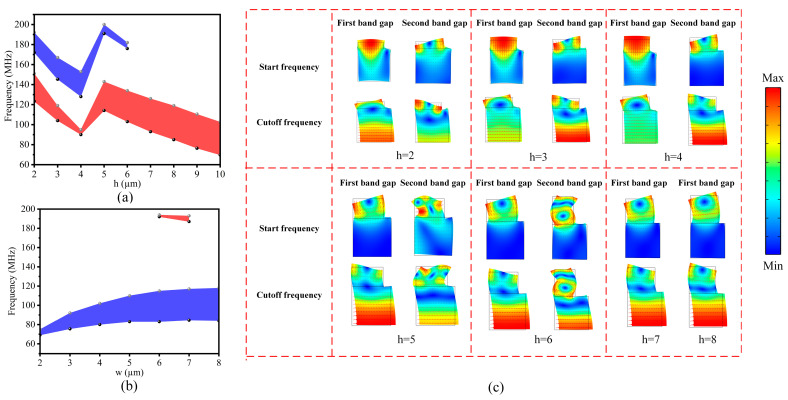
(**a**) The effect of the metal height h on the band gap. The red portion represents the first band gap and the blue color represents the second band gap. (**b**) The effect of the metal width w on the band gap. The blue portion represents the first band gap and the red portion represents the second band gap. (**c**) The eigenmodes of the starting frequency and cutoff frequency of the band gap from 2 µm to 8 µm for h.

In the design of the “L”-shaped M-RPC structure, the effect of the metal height h on the band-gap range was investigated by keeping the lattice constant a, the silicon plate height H, and the metal width w constant, as shown in Figure 7a. When h increased from 2 µm to 4 µm, the start and cutoff frequencies of the first band gap (the red part) and the second band gap (the blue part) gradually fell to lower frequencies, and the first band-gap bandwidth decreased while the second band-gap bandwidth gradually increased. When h was equal to 5 µm, the first band gap and the second band gap rose to high frequencies again, and the first band gap became wider while the second band gap became narrower. When h increased from 5 µm to 6 µm, the first band gap and the second band gap again gradually moved to lower frequencies, and their bandwidths increased and decreased, respectively. After h grew larger than 7 µm, a band gap was created that moved to lower frequencies, and its band-gap width remained unchanged. Under the precondition of guaranteeing the band-gap width, the smaller the metal height h was, the smaller the mass of the structure was, which also met the goal of structural lightweighting, and so this study chose the metal height h to be 8 µm. As shown in Figure 7a, discontinuity occurred when h changed from 4 µm to 5 µm. In order to analyze the reasons, the eigenmodes at the starting frequency and cutoff frequency of the band gap were calculated when h changed from 2 µm to 8 µm, as shown in Figure 7c. It could be seen that when h changed from 2 µm to 4 µm, the eigenmodes of the starting frequency and cutoff frequency of the band gap remained consistent, whereas when it changed from 4 µm to 5 µm, the eigenmodes of the starting and cutoff frequencies of the first and second band gaps changed compared to the previous ones, and this was the reason for the discontinuity in the band-gap width between h = 4 µm and h = 5 µm.

The effect of the metal width w on the band gap of the M-RPC structure was investigated by keeping the lattice constant a, the silicon plate height H, and the metal height h constant. From Figure 7b, it can be seen that when the metal width w was gradually increased from 2 µm to 8 µm, both the start and cutoff frequencies of the first band gap (the blue color) gradually increased, and the cutoff frequency grew faster than the start frequency, and so the band gap range became wider gradually. The second narrower band gap (the red part) was generated when the metal widths were 6 µm and 7 µm, respectively. The best band-gap width was available when h was equal to 8 µm.

## 4. Resonator Design and Analysis Results

### 4.1. Resonator Design

**Figure 8:**
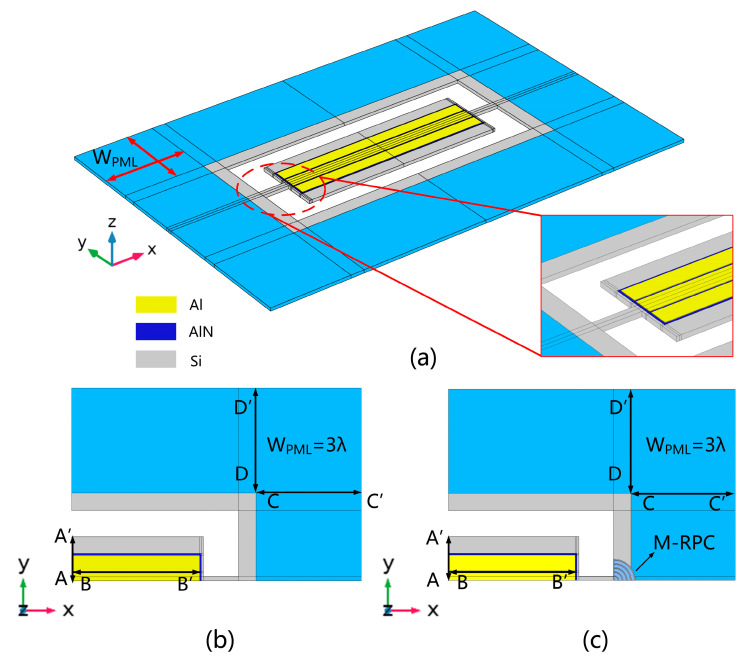
Piezoelectric MEMS resonator model. (**a**) A 3D model of the piezoelectric MEMS resonator. (**b**) A quarter model of a conventional piezoelectric MEMS resonator. (**c**) A quarter model of an M-RPC piezoelectric MEMS resonator.

Since the M-RPC structure generated a band gap range of 84.1–118.3 MHz, acoustic propagation and mechanical vibration were prohibited [12,14]. Therefore, in this study, a fifth-order piezoelectric MEMS resonator with a resonant frequency of 100 MHz was designed to attenuate the energy that leaked from the anchor point, and thus, it improved the quality factor of the resonator. Figure 8a shows a 3D model of the piezoelectric MEMS resonator. The outer blue part of the model indicates the PML layer. Due to the symmetry of the piezoelectric MEMS resonator structure, only one quarter of the structure was modeled in the simulation. Figure 8b shows a quarter-simplified model of a conventional piezoelectric MEMS resonator, and Figure 8c shows a quarter-simplified model of an M-RPC piezoelectric MEMS resonator composed of Pt and Si.

The expression for the width-extended (WE) mode of vibration of the resonator is given as follows [30,31,32]:

(4) $$f_{r}=\frac{nv}{2W_{r}},$$

where n is the order of the resonant mode, v is the sound velocity of the corresponding resonant mode, and $W_{r}$ is the width of the resonator. The specific design dimensional parameters of the resonator are shown in Table 2, and the material parameters used for the resonator are shown in Table 3.

### 4.2. Analysis Results

In order to verify the effectiveness of using the M-RPC structure to reduce the anchor loss of the piezoelectric MEMS resonator, the finite element method was used to calculate the $Q_{anchor}$ value of the resonator. The value of $Q_{anchor}$ could be obtained as follows [33]:

(5) $$Q_{anchor}=\frac{Re(f)}{2Im(f)},$$

where f is the resonant frequency of the resonator, Re(f) represents the real part of the resonant frequency, and Im(f) represents the imaginary part of the resonant frequency.

**Figure 9:**
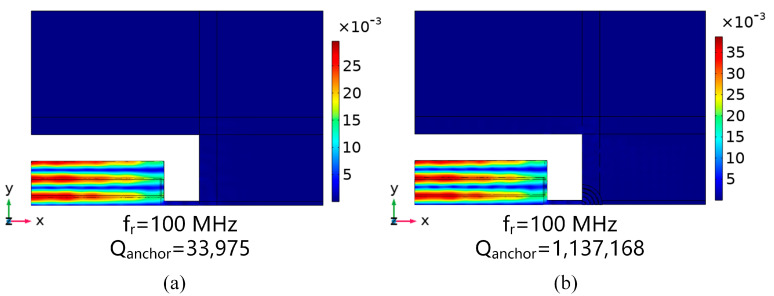
(**a**) Schematic diagram of the total displacement distribution of the conventional piezoelectric MEMS resonator at 100 MHz. (**b**) Schematic diagram of the total displacement distribution of the “L”−type M−RPC piezoelectric MEMS resonator at 100 MHz.

**Figure 10:**
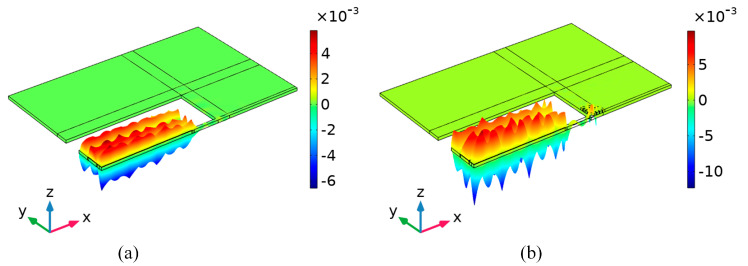
(**a**) Schematic diagram of the z−direction displacement distribution of the conventional piezoelectric MEMS resonator at 100 MHz. (**b**) Schematic diagram of the z−direction displacement distribution of the “L”−type M-RPC piezoelectric MEMS resonator at 100 MHz.

Figure 9a,b shows the total displacement distributions of the conventional piezoelectric MEMS resonator and the M-RPC piezoelectric MEMS resonator at 100 MHz, respectively. The total displacement field at the resonance of the “L”-type M-RPC resonator was larger than that of the conventional piezoelectric MEMS resonator, indicating that the mechanical energy loss of the M-RPC resonator was smaller. Moreover, the $Q_{anchor}$ value of the M-RPC resonator (1,137,168) was 33.5 times higher than that of the conventional resonator (33,975), as can be seen from Figure 9. The vibration modes of the conventional piezoelectric MEMS resonator and the M-RPC piezoelectric MEMS resonator in the z-axis direction are plotted in Figure 10a and Figure 10b, respectively. By adjusting the scale factor of the finite element simulation to increase its deformation amplitude and observing the displacement amplitude in the z-direction, it could be seen that the amplitude of the “L” type M-RPC resonator was larger in the resonator, which indicated that the anchor loss of the Lamb wave through the M-RPC resonator was smaller.

**Figure 11:**
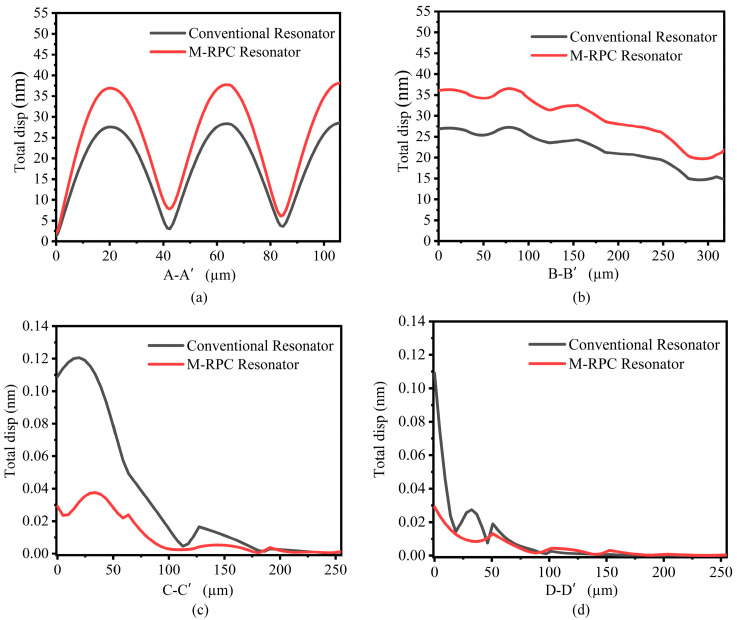
(**a**) The total displacement along the resonator A-A′ at 100 MHz. (**b**) The total displacement along the resonator B-B′ at 100 MHz. (**c**) The total displacement along the resonator C-C′ at 100 MHz. (**d**) The total displacement along the resonator D-D′ at 100 MHz.

In order to quantitatively analyze the resonance effects of the conventional piezoelectric MEMS resonator and the M-RPC resonator, the total displacements were plotted along the A-A’, B-B′, C-C′, and D-D′ lines (see Figure 8), respectively, as shown in Figure 11. From Figure 11a,b, it can be seen that the displacements at the resonance site of the proposed “L”-type M-RPC piezoelectric MEMS resonator were larger compared with those of the conventional resonator, indicating that the energy stored in the M-RPC resonator was much greater than that of the conventional resonator. From Figure 11c,d, it can be seen that the M-RPC resonator had a smaller displacement than the conventional resonator, which indicated that less energy was leaked when the Lamb wave passed through the support beam of the M-RPC resonator.

**Figure 12:**
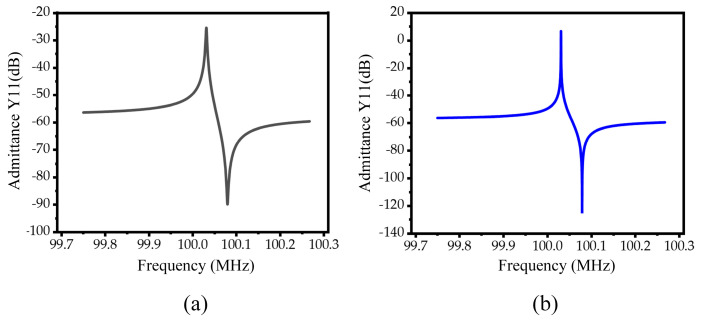
(**a**) Conventional piezoelectric MEMS resonator admittance curve diagram. (**b**) M−RPC piezoelectric MEMS resonator admittance curve diagram.

In order to further analyze the performance of the designed resonators, the finite element method was used to simulate the resonators under a 50 Ω impedance-matching, and the admittance curve was obtained, as shown in Figure 12. The electromechanical coupling coefficient of piezoelectric MEMS resonators can be calculated by Formulas (6) [34] as follows:

(6) $${K^{2}}_{eff}=\frac{f_{p}^{2}-f_{s}^{2}}{f_{p}^{2}},$$

where $f_{p}$ is the frequency at which the impedance amplitude is at its maximum and $f_{s}$ is the frequency at which the impedance amplitude is at its minimum.

**Figure 13:**
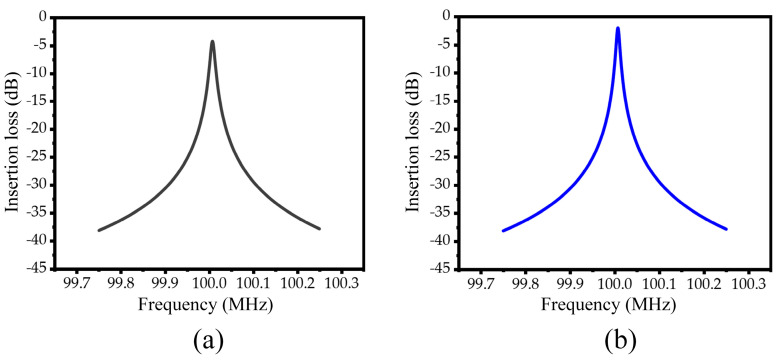
(**a**) Conventional piezoelectric MEMS resonator insertion-loss curve diagram. (**b**) M−RPC piezoelectric MEMS resonator insertion-loss curve diagram.

Figure 13a,b shows the insertion loss curves of the conventional piezoelectric MEMS resonator and the M-RPC piezoelectric MEMS resonator, respectively. The loaded quality factor ( $Q_{l}$ ) and the unloaded quality factor ( $Q_{u}$ ) could be calculated, respectively, by Equation (7) and Equation (8) [9,16] as follows:

(7) $$Q_{l}=\frac{f_{s}}{\Delta f_{-3dB}}\;\mathrm{and}$$

(8) $$Q_{u}=\frac{Q_{l}}{1-10^{-\frac{IL}{20}}},$$

where $\Delta f_{-3dB}$ is the −3 dB bandwidth and *IL* is the insertion loss.

When the conventional resonator was used, the value of $Q_{l}$ was 9902, the value of $Q_{u}$ was 25,826, the insertion loss was 4.2 dB, and the effective electromechanical coupling coefficient was 0.12%. When the M-RPC resonator was used, the value of $Q_{l}$ was 17,395, the value of $Q_{u}$ was 86,505, the insertion loss was 1.95 dB, and the effective electromechanical coupling coefficient was 0.12%. Compared with the conventional resonator, the M-RPC resonator had improved the values of $Q_{l}$ and $Q_{u}$ by 75.7% and 235.0%, respectively, and the insertion loss had been reduced from 4.2 dB to 1.95 dB. The effective electromechanical coupling coefficient had not been affected and remained 0.12%. The specific performance parameters of the conventional piezoelectric MEMS resonator and the M-RPC piezoelectric MEMS resonator are shown in Table 4.

## 5. Conclusions

In this paper, an “L”-type M-RPC structure is proposed to reduce the anchor-point loss and improve the quality factor of piezoelectric MEMS resonators.

Firstly, the dispersion curve and frequency transmission response of the M-RPC structure were calculated using the finite element method. Compared with the S-RPC structure, the M-RPC structure could generate a band gap in the range of 84.1–118.3 MHz. When the frequency was 88 MHz, the maximum attenuation reached 63 dB. Then, when different metal materials were combined with Si in different RPC structures, it was found that the larger the mass densities and Young’s moduli parameter differences were between the metal and silicon, the larger the widths of the complete band gaps were. Further, the effects of the height h and width w of the metal Pt on the band gaps were analyzed to obtain a better band gap. Finally, by applying the M-RPC structure to a piezoelectric MEMS resonator, the anchor quality factor was increased from 33,975 to 1,137,168, and the insertion loss was reduced from 4.2 dB to 1.95 dB compared to the conventional resonator while the loaded and unloaded quality factors were increased from 9902 and 25,826 to 17,395 and 86,505, respectively. In addition, the addition of the M-RPC structure had no effect on the electromechanical coupling coefficient, which remained 0.12%.

## Figures and Tables

**Table 1:** Parameters of the different metal materials.

Materials	Young’s Modulus (GPa)	Density (kg/m^3^)
Copper (Cu)	120	8960
Aluminum (Al)	70	2700
Lead (Pd)	73	12,020
Platinum (Pt)	168	21,450

**Table 2:** Design dimensional parameters of the resonators.

Parameters	Values (Unit)
Resonator resonant frequency ( $f_{r}$ )	100 (MHz)
Wavelength ( λ )	84.8 (µm)
Inter-digitated transducer (IDT) finger ( n )	5
Tether width (W_t_)	20 (µm)
Tether length (L_t_)	84.8 (µm)
Electrode gap (G_e_)	4 (µm)
Resonant width (W_r_)	212 (µm)
Resonant length (L_r_)	636 (µm)
Thickness of the Al (T_Al_)	1 (µm)
Thickness of the AlN (T_AlN_)	0.5 (µm)
Thickness of the substrate Si (T_Si_)	10 (µm)

**Table 3:** Resonator material characteristics.

Materials	Parameters	Values
Aluminum nitride (AIN)	Mass density ( ρ )	3300 kg/m^3^
	Young’s modulus (E)	320 GPa
	Poisson’s ratio (ν)	0.24
	Relative permittivity (ε)	9
Aluminum (Al)	Mass density ( ρ )	2700 kg/m^3^
	Young’s modulus (E)	70 GPa
	Poisson’s ratio (ν)	0.35
	Electrical conductivity (σ)	35.5 × 10^6^ S/m
	Coefficient of thermal expansion (α)	23.1 × 10^−6^ 1/K
	Heat capacity (Cp)	904 J/(Kg K)
	Thermal conductivity (κ)	237 W/(mK)
Silicon (Si)	Mass density ( ρ )	2330 kg/m^3^
	Young’s modulus (E)	E_x_ = 169 GPa
		E_y_ = 169 GPa
		E_z_ = 130 GPa
	Shearing’s modulus (G)	G_xy_ = 50.9 GPa
		G_yz_ = 79.6 Gpa
		G_zx_ = 79.6 Gpa
	Poisson’s ratio (ν)	σ_xy_ = 0.064
		σ_yz_ = 0.36
		σ_zx_ = 0.28

**Table 4:** Comparison of the performance parameters of the two resonators.

Parameters	Conventional Resonator	M-RPC Resonator
$\mathrm{Resonant}\;\mathrm{frequency}\;(f_{r}$ ), MHz	100	100
Insertion loss (IL ), dB	4.2	1.95
$\mathrm{Coupling}\;\mathrm{coefficient}\;(k_{eff}^{2}$ ), %	0.12	0.12
$\mathrm{Simulated}\;Q_{anchor}$	33,975	1,137,168
$\mathrm{Loaded}\;\mathrm{quality}\;\mathrm{factor}\;(Q_{l}$ )	9902	17,395
$\mathrm{Unloaded}\;\mathrm{quality}\;\mathrm{factor}\;(Q_{u}$ )	25,826	86,505

## Data Availability

All data needed to evaluate the conclusions in the paper are presented in the paper.
